# Support for e-cigarette regulations among Australian young adults

**DOI:** 10.1186/s12889-019-6410-4

**Published:** 2019-01-15

**Authors:** Michelle I. Jongenelis, Caitlin Kameron, Daniel Rudaizky, Simone Pettigrew

**Affiliations:** 1 School of Psychology, Curtin University, Kent Street, Bentley, Western Australia 6102 Australia; 2 Cancer Council WA, 420 Bagot Road, Subiaco, Western Australia 6008 Australia; 3 School of Psychological Science, The University of Western Australia, 35 Stirling Highway, Crawley, Western Australia 6009 Australia

**Keywords:** E-cigarettes, Regulation, Policy, Harms, Young adults

## Abstract

**Background:**

Surveying support for various regulatory options relating to e-cigarettes can assist policymakers to identify those that have broad support and are therefore likely to be easier to implement. However, data on support for potential e-cigarette regulations in Australia are limited. To inform regulatory efforts, the present study assessed attitudes to the regulation of e-cigarettes among Australian young adults, the most prevalent users of e-cigarettes and therefore the most likely population segment to be affected by e-cigarette regulations.

**Methods:**

A total of 1116 Australians aged 18 to 25 years (59% female) completed an online survey where they were presented with various statements relating to the regulation of e-cigarettes and asked to report on the extent to which they agreed or disagreed with each. Statements presented either a restrictive or non-restrictive approach to e-cigarette regulation.

**Results:**

Across all statements, 10–22% of respondents responded “don’t know” while 23–35% neither agreed nor disagreed, indicating general ambivalence. There was a moderate level of support (33–37%) for regulating e-cigarette sales/use and treating e-cigarettes like tobacco products. Only 20% of respondents were in favour of allowing the use of e-cigarettes in smoke-free areas. Smokers, e-cigarette users, and those who did not believe in the harms associated with e-cigarettes were typically less likely than other respondents to support restrictive approaches.

**Conclusions:**

The young Australian adults surveyed were somewhat supportive of restrictions around the sale and use of e-cigarettes, but generally opposed outright bans and any need for a prescription from a medical practitioner. Increasing awareness of the harms associated with the use of e-cigarettes represents a potential strategy to gaining regulatory support.

**Electronic supplementary material:**

The online version of this article (10.1186/s12889-019-6410-4) contains supplementary material, which is available to authorized users.

## Background

Recent years have seen an increase in the popularity of electronic nicotine delivery systems, especially electronic cigarettes (or e-cigarettes) [1, 2]. In Australia, the context of the present study, figures from the recent National Drug Strategy Household Survey (NDSHS) show an increase in lifetime use of e-cigarettes among adults from 4% in 2013 to 9% in 2016 [3]. This increase was observed in both smokers (18 to 31%) and non-smokers (2 to 5%). By contrast, tobacco cigarette smoking rates have decreased over time and this has been attributed to tobacco control policies such as taxation, clean air laws, advertising restrictions on tobacco products, product labelling, and the denormalisation of smoking behaviours [4, 5].

The substantial growth in the use of e-cigarettes has prompted calls for regulation of these devices to (i) minimise potential health risks to both users and non-users (via ‘passive vaping’) and (ii) prevent the renormalisation of smoking behaviours [6, 7]. Presently, the legal status of e-cigarettes varies widely across countries and jurisdictions [8, 9]. In Australia, the legal status of e-cigarettes is determined by existing and overlapping laws relating to poisons, therapeutic goods, consumer goods, and tobacco control. Generally, different laws apply depending on whether e-cigarettes contain liquid nicotine [10]. Nicotine is classified as a dangerous poison under Schedule 7 of the *Australian Standard Uniform Scheduling of Medicines and Poisons* and, as such, the manufacture, sale, or supply of e-cigarettes containing nicotine without lawful authority is prohibited in all Australian states and territories. However, individual users are able to lawfully purchase nicotine-containing e-cigarettes from overseas for personal use provided (i) they hold a valid prescription from a registered Australian medical practitioner and (ii) possession and use of an e-cigarette containing nicotine is legal within the user’s state or territory [11, 12]. Individual users are also able to unlawfully obtain nicotine-containing e-cigarettes and it has been suggested that a substantial black market for these types of e-liquids likely exists in Australia [10]. There is also evidence to suggest that a substantial majority of the e-liquids sold in Australia are incorrectly labelled, with scientific testing revealing the presence of high levels of nicotine in e-liquids that did not have nicotine listed as an ingredient [13]. This leads to the potential for consumers to be misinformed about the products they are consuming and may result in excessive nicotine intake and subsequent adverse health effects.

Nicotine is only one of the many toxic ingredients found in e-cigarettes, with tests of e-liquids demonstrating the cytotoxicity of non-nicotine ingredients such as additives and flavourings [14, 15]. These toxins impact the quality of indoor environments and expose users to additional risk factors for cardiovascular disease and cancer [16–19]. Despite these risks, non-nicotine e-cigarettes are not uniformly regulated, which means the laws surrounding their sale and use in Australia are determined by individual states and territories. At the time this study was conducted (August – September 2016), only three of the eight Australian states and territories had specifically regulated the sale and use of e-cigarettes. Since this study was conducted, more jurisdictions have extended their tobacco control legislation to include specific provisions around the advertising, sale, and use of e-cigarettes. However, given legislation continues to vary between states and territories, one of the recommendations of a recent Parliamentary Inquiry into the use of e-cigarettes in Australia included the adoption of a national approach to the regulation of non-nicotine e-cigarettes to ensure regulations are applied consistently across all jurisdictions [20]. The Inquiry also recommended the establishment of a regulatory process to assess and restrict the non-nicotine constituents of e-cigarettes.

Support for policies is considered important to their successful implementation [21]. As such, the Inquiry’s recommendation for a clear regulatory framework with nationally-mandated rules for the sale, purchase, possession, and use of both nicotine and non-nicotine-containing e-cigarettes makes it important to gauge public opinion. However, data on support for potential e-cigarette policies in Australia are limited. The vast majority of research assessing public support for e-cigarette policies has been conducted in the US and the UK where there are less restrictive regulatory environments [10, 22]. Results from these studies suggest that most people are supportive of stricter regulation of e-cigarettes [23, 24]. Support for such policies tends to be stronger among non-smokers relative to smokers of traditional cigarettes [23, 24]. Research also suggests that tobacco smokers’ and ex-smokers’ perceptions of the harm associated with e-cigarettes relative to traditional cigarettes moderate support for various e-cigarette policies, with those who believe e-cigarettes to be as harmful or more harmful than tobacco cigarettes more likely to support restrictive policies [23, 25].

Whether policy support is moderated by perceptions of the absolute harm associated with e-cigarette use (rather than just the relative harm compared to traditional cigarettes) does not appear to have been assessed, nor have the views of non-smokers towards e-cigarette policy initiatives. In the one study conducted in Australia to date, the sample comprised e-cigarette users only [26]. Most of the study participants did not want restrictions placed on their ability to access and use e-cigarettes, which is consistent with other research indicating that differences in policy support are moderated by e-cigarette user status [23, 25].

## Present study

Surveying opinions of various regulations relating to e-cigarettes can assist in the successful implementation of future policies and can help policymakers identify policies that (i) have broad support and are therefore likely to be easier to implement and (ii) are not generally supported and may benefit from efforts to build support to facilitate successful implementation [27]. To inform regulatory efforts in Australia, the aim of the present study was to assess young adults’ attitudes to various statements relating to the regulation of e-cigarettes. Differences in opinion by gender, age, smoking status, e-cigarette user status, and harm perceptions (both absolute and relative) were also assessed.

Young adults were of specific interest because e-cigarettes are considered especially appealing to this population segment, as evidenced by higher prevalence of use. For example, in Australia 49% of smokers and 14% of non-smokers aged 18 to 24 years had used an e-cigarette in their lifetime compared to 31 and 5% respectively among the general adult population [3]. The higher prevalence of use among youth, especially non-smoking youth, has prompted concerns about the potential for e-cigarettes to act as a gateway to traditional smoking [28] and calls have been made for greater regulation of e-cigarettes in Australia to account for the potential for unintended harm among youth [10, 29]. Previous research also suggests the majority of young adults use e-cigarettes for recreational reasons or out of curiosity rather than for therapeutic purposes [30]. By contrast, studies of the general population indicate that the majority of individuals cite quitting or reducing smoking as reasons for use [31, 32]. The views of young adults are therefore especially important to examine because (i) their status as the most prevalent users of e-cigarettes means they are more likely to be directly affected by e-cigarette regulations and (ii) their support for various policy options may differ to those of the general population as a result of their differing reasons for use.

## Method

### Sample

Approval to conduct this study was obtained from Curtin University’s Human Research Ethics Committee and written informed consent was obtained from all respondents. The sample was recruited via PureProfile, an ISO-accredited web panel provider with access to a database of geographically and socioeconomically diverse Australians. The database was established and is replenished using strategies such as internet and radio advertising and referrals. The sample comprised 1116 Australians aged 18 to 25 years (*M* = 21.56, *SD* = 2.32, 59% female) who were participating in an online study examining e-cigarette use among young adults. A quarter of respondents (25%) were current smokers [i.e., reported smoking > 100 tobacco cigarettes in their lifetime and had smoked a tobacco cigarette in the last 30 days as per [22, 33] and nearly half (47%) were never smokers. Remaining respondents had smoked in the past but not recently and were therefore classified as former smokers (28%). The proportion of current smokers in the present sample was greater than the 17% obtained in the population-representative 2016 NDSHS [3]. Current use of e-cigarettes (i.e., use at a frequency of at least monthly and use within the last 30 days) was reported by 9% of respondents. This proportion is similar to that obtained in the NDSHS (7%).

### Measures

The measures used in the present study can be viewed in the online Additional file 1. After answering questions relating to their tobacco and e-cigarette use (e.g., frequency of use, reasons for initiation, reasons for continued use), respondents were presented with various statements relating to the regulation of e-cigarettes and asked to report on the extent to which they agreed or disagreed with each on a scale of 1 (*strongly disagree*) to 5 (*strongly agree*). A “don’t know” option was also provided. Statements presented either a restrictive (e.g., *E-cigarettes should be treated as if they were prescription medicines*) or non-restrictive (e.g., *You should be able to use e-cigarettes in places that do not allow smoking*) approach to the regulation of e-cigarettes.

Absolute harm perceptions were assessed by asking respondents to indicate how harmful they believe e-cigarettes are to health (1 = *not at all harmful* to 5 = *very harmful*, 6 = *don’t know*). Relative harm perceptions were assessed by asking respondents to indicate whether they believed e-cigarettes to be less harmful, equally harmful, or more harmful than tobacco cigarettes. A “don’t know” option was also provided.

### Statistical analyses

Descriptive analyses were conducted to assess support for each of the presented statements. Independent samples *t*-tests were used to examine differences in support by gender, age, and e-cigarette user status. Age was dichotomised into the categories of 18–19 year olds and 20–25 year olds to ensure consistency with the reporting of e-cigarette use in the NDSHS. One-way ANOVAs with Fisher’s LSD post hoc tests were conducted to examine differences in support by smoking status. As some respondents were both current smokers and e-cigarette users, these categories were not mutually exclusive. To test whether any significant findings by smoking status were being driven by e-cigarette user status, sensitivity analyses stratifying findings by e-cigarette user subgroup (i.e., user, non-user) were conducted.

The association between absolute harm perceptions and statement support was assessed using correlation analyses. Partial correlation analyses were also performed adjusting for e-cigarette user status. Independent samples *t*-tests were used to assess the association between relative harm perceptions (0 = believe e-cigarettes to be less harmful or equally harmful to tobacco cigarettes, 1 = believe e-cigarettes to more harmful than tobacco cigarettes) and statement support. For all parametric analyses involving the assessment of group differences, those responding “don’t know” were treated listwise.

## Results

Respondents’ support for various statements relating to the regulation of e-cigarettes is presented in Table 1. Across all statements, 10–22% of respondents answered “don’t know” and 23–35% neither agreed nor disagreed. Statements presenting restrictive regulations failed to achieve majority support in all instances. Of these, support was greatest (albeit moderate) for treating e-cigarettes like tobacco products, with around one in three respondents agreeing with this statement. The statements suggesting that e-cigarettes should be treated like prescription medicines and that the supply of non-nicotine e-cigarettes should be prohibited were least supported, with just one in ten agreeing with these statements. Of the two statements presenting non-restrictive regulations, allowing the use of e-cigarettes in smoke-free areas was the least supported (one in five respondents).

**Table 1:** Opinions related to the regulation of e-cigarettes in the overall sample and stratified by gender and age

Statement				Gender						Age					
	Overall			Males			Females			18–19 year olds			20–25 year olds		
	*N* = 1116			*n* = 459			*n* = 657			*n* = 267			*n* = 849		
	M (*SD*)	A %	D%	M (*SD*)	A %	D%	M (*SD*)	A%	D%	M (*SD*)	A%	D%	M (*SD*)	A%	D%
Restrictive policies															
Treat like tobacco cigarettes	3.24 (1.10)	37^a^	20	3.14* (1.16)	35^a^	24	3.31 (1.06)	38^a^	18	3.21 (1.09)	35^a^	21	3.25 (1.11)	37^a^	20
Treat like prescription medicines	2.62 (1.06)	15^a^	35	2.63 (1.11)	18^a^	38	2.62 (1.02)	13^a^	33	2.63 (1.03)	15^a^	37	2.62 (1.07)	15^a^	35
Prohibit supply of nicotine e-cigarettes	2.98 (1.04)	21	22	2.95 (1.14)	25	27	3.00 (0.96)	19	19	2.95 (1.03)	20	21	2.99 (1.05)	22	23
Prohibit supply of non-nicotine e-cigarettes	2.72 (1.00)	13^a^	29	2.75 (1.11)	19^a^	32	2.70 (0.91)	9^a^	28	2.59* (0.93)	9^a^	30	2.76 (1.02)	15^a^	29
Only sold in pharmacies	2.91 (1.07)	23	26	2.95 (1.12)	26	25	2.88 (1.04)	22	27	2.86 (1.09)	23	30	2.93 (1.07)	24	25
Non-restrictive policies															
Available as an over the counter purchase at regular shops	2.88 (1.12)	25	28	2.92 (1.16)	27	29	2.85 (1.08)	23	28	2.77 (1.08)	20^a^	30	2.91 (1.13)	26	28
Should be able to use in smoke-free places	2.45 (1.22)	20^a^	48	2.51 (1.26)	22^a^	46	2.41 (1.19)	18^a^	50	2.33 (1.21)	18^a^	53	2.49 (1.22)	20^a^	47

*A* agree, *D* disagree. Those responding “don’t know” were excluded from the calculation of means but included in the calculation of percentages

^a^Significantly different to disagree; *Significantly different from relevant comparison group at *p* < .05

### Gender and age differences

Gender and age differences in support for various statements relating to the regulation of e-cigarettes are presented in Table 1. Results were largely consistent with those found in the overall sample. A significant gender difference was observed for the statement suggesting that e-cigarettes should be treated like tobacco cigarettes: females were more likely than males to agree with this statement. A significant age difference was observed for the statement suggesting that the supply of non-nicotine e-cigarettes should be prohibited: 18 to 19 year olds were less likely than 20 to 25 year olds to agree with this statement.

### Smoking status differences

Smoking status differences in support for various statements relating to the regulation of e-cigarettes are presented in Table 2. One-way ANOVAs with Fisher’s LSD post hoc tests revealed a significant smoking status difference for three of the seven statements. Current smokers were more likely than former smokers and never smokers to agree that e-cigarettes should be readily available as an over the counter purchase at regular shops and allowed to be used in places that do not allow smoking, but less likely to agree that e-cigarettes containing nicotine should be prohibited. Current smokers were also less likely than never smokers to agree that e-cigarettes should only be sold in pharmacies. Analyses stratifying by e-cigarette user status revealed that these significant differences by smoking status were only observed in non-users of the devices.

**Table 2:** Opinions related to the regulation of e-cigarettes stratified by smoking status and e-cigarette use status

Statement	Smoking status									E-cigarette use status					
	Smokers			Former smokers			Never smokers			User			Non-user		
	*n* = 272			*n* = 316			*n* = 519			*n* = 104			*n* = 1012		
	M (*SD*)	A %	D %	M (*SD*)	A%	D %	M (*SD*)	A %	D %	M (*SD*)	A %	D %	M (*SD*)	A %	D %
Restrictive policies															
Treat like tobacco cigarettes	3.15 (1.11)	34^a, c^	23	3.27 (1.03)	39^a^	20	3.26 (1.15)	37^a^	19	3.24 (1.14)	40^a^	24	3.24 (1.10)	36^a^	20
Treat like prescription medicines	2.56 (1.09)	15^a, c^	40	2.60 (0.97)	13^a^	35	2.68 (1.10)	17^a^	33	2.93* (1.28)	35	35	2.59 (1.02)	13^a^	35
Prohibit supply of nicotine e-cigarettes	2.75^bc^ (1.06)	16^a, c^	33	2.98 (0.96)	21	21	3.12 (1.06)	25^a^	17	3.08 (1.12)	35	28	2.97 (1.03)	20	22
Prohibit supply of non-nicotine e-cigarettes	2.71 (1.07)	14^a, c^	32	2.68 (0.92)	12^a^	33	2.76 (1.01)	14^a^	25	3.03* (1.24)	35	32	2.68 (0.96)	11^a^	29
Only sold in pharmacies	2.78^c^ (1.10)	20^a, c^	33	2.90 (0.97)	22	25	2.99 (1.12)	26	23	3.26** (1.23)	44^a^	26	2.87 (1.04)	21^a^	26
Non-restrictive policies															
Available as an over the counter purchase at regular shops	3.29^bc^ (1.10)	40^a, c^	19	2.99^c^ (1.02)	27	25	2.57 (1.10)	15^a^	35	3.54*** (1.09)	51^a^	14	2.80 (1.09)	22^a^	30
Should be able to use in smoke-free places	3.35^bc^ (1.45)	34	31	2.87^c^ (1.55)	21^a^	47	2.46 (1.54)	12^a^	57	3.23*** (1.29)	48	32	2.36 (1.18)	17^a^	50

*A* agree, *D* disagree. Those responding “don’t know” were excluded from the calculation of means but included in the calculation of percentages

^a^Significantly different to disagree; ^b^Significantly different to former smokers; ^c^Significantly different to never smokers

*Significantly different from non-users at *p* < .05; **Significantly different from non-users at *p* < .01; ***Significantly different from non-users at *p* < .001

### User-status differences

User-status differences in support for various statements relating to the regulation of e-cigarettes are presented in Table 2. Independent samples *t*-tests conducted to assess for differences by e-cigarette user status revealed that users were more likely than non-users to believe that e-cigarettes should be (i) made readily available as an over the counter purchase at regular shops, (ii) allowed in places that do not allow smoking, (iii) treated as if they are prescription medicines, and (iv) only sold in pharmacies like other non-cigarette products that contain nicotine. In addition, users were more likely than non-users to believe that the supply of e-cigarettes that do not contain nicotine should be prohibited.

### Harm perceptions

Correlations between respondents’ perceptions of the absolute harm associated with e-cigarette use and opinion towards the regulation of e-cigarettes are presented in Table 3. Greater perceived harm was associated with greater support for restrictive policies, with these correlations small to moderate in size. This pattern of results remained when adjusting for e-cigarette user status.

**Table 3:** Correlations between absolute harm perceptions and opinions related to the regulation of e-cigarettes

Statement	Absolute harm perceptions	Absolute harm perceptions (adjusted for user status)
Treated like tobacco cigarettes	.22***	.25***
Prohibit supply of nicotine e-cigarettes	.17***	.21***
Only sold in pharmacies like other non-cigarette products containing nicotine	.05	.03
Prohibit supply of non-nicotine e-cigarettes	.14***	.18***
Treated like prescription medicines	−.04	.02
Readily available as an over the counter purchase at regular shops	−.28***	−.25***
Should be able to use in smoke-free places	−.29***	−.28***

Those responding “don’t know” were treated listwise

***p < .001

Results pertaining to relative harm perceptions were similar to those obtained for absolute harm perceptions. Those who believed e-cigarettes to be more harmful were (i) more likely to believe that the supply of nicotine (*t*(347.82) = − 4.61, *p* < .001, *d* = − 0.39) and non-nicotine (*t*(709) = − 3.94, *p* < .001, *d* = − 0.31) e-cigarettes should be prohibited and that e-cigarettes should be treated like tobacco products (*t*(399.85) = − 3.89, *p* < .001, *d* = − 0.31) and (ii) less likely to believe that e-cigarettes should be made readily available at regular shops (*t*(403.63) = 5.66, *p* < .001, *d* = 0.46) and allowed in places that do not allow smoking (*t*(798) = 5.16, *p* < .001, *d* = 0.39).

## Discussion

Given the importance of public support for successful policy implementation [21], the present study aimed to survey Australian young adults’ attitudes towards various options for the regulation of e-cigarettes. The young adults surveyed in the present study were generally ambivalent in their attitudes to the various options, with 32 to 58% of respondents selecting the “don’t know” or “neither agree nor disagree” response options for each of the statements. Such responses are reflective of the continued uncertainty and contradictory messages within the scientific community regarding the benefits and risks of e-cigarette use [34].

Moderate levels of support were observed for the treatment of e-cigarettes as a tobacco product, with significantly more young adults supporting than opposing this statement. By contrast, the use of e-cigarettes in places that do not allow smoking was the most strongly opposed, with nearly half of all respondents believing that use of e-cigarettes in such areas should be prohibited. This may at least partially reflect an increase in public concern over the risks associated with passive exposure to e-cigarette vapour [27]. The introduction of a policy prohibiting e-cigarette use in smoke-free areas is important for a number of reasons. First, e-cigarette initiation is facilitated by the ability to use e-cigarettes in places where smoking is prohibited [32, 35, 36]. The introduction of a policy prohibiting use in these areas therefore has the potential to result in a reduction in the uptake of e-cigarettes.

Second, the significant gains made with respect to tobacco control are due in part to the introduction of smoke-free policies that make it difficult for smokers to use cigarettes, and it has been suggested that smokers may use e-cigarettes to circumvent smoke-free laws rather than attempting cessation [37–39]. Third, research indicates that seeing an individual use an e-cigarette increases smokers’ urge to smoke traditional cigarettes [40]. Finally, the significant gains made with respect to tobacco control are at least partly due to the denormalisation of smoking behaviours [5]. Concerns have been raised that the use of e-cigarettes in places that do not allow smoking will result in renormalisation of such behaviours [5, 41, 42]. The use of e-cigarettes in smoke-free areas therefore has the potential to hinder cessation attempts, increase smoking among current smokers, and lead to relapse among ex-smokers. The introduction of legislation prohibiting the use of e-cigarettes in places that do not allow traditional smoking may serve to mitigate these risks.

A substantial minority of respondents opposed the statements suggesting that e-cigarettes should be treated as prescription medicines and that non-nicotine e-cigarettes should be prohibited, suggesting that the introduction of these policies may be met with resistance from some members of this age group. The low levels of support for the treatment of e-cigarettes as prescription medicines is not surprising given previous research suggests the majority of young adults use e-cigarettes out of curiosity and for recreational rather than therapeutic purposes [30], and therefore may be unable to obtain a prescription. Among those using e-cigarettes for therapeutic purposes, the need to obtain a prescription and therefore visit a registered medical practitioner may be perceived as an overly burdensome, onerous, and financially costly process [43].

The particularly low levels of support for the statement that non-nicotine e-cigarettes should be prohibited indicates that efforts may be needed to increase support for the implementation of any regulations related to these devices, such as those recommended by the Parliamentary Inquiry into the use of e-cigarettes in Australia [20]. Given that greater belief in the harms associated with e-cigarette use was found to be associated with support for restrictive regulation of e-cigarettes, increasing awareness of the harms associated with the use of e-cigarettes and challenging beliefs of e-cigarette use as a risk-free habit represent potential strategies to gain regulatory support.

Consistent with the proposition that preferences for public policies are determined by people’s self-interest [44], current smokers and e-cigarette users were typically less likely than other respondents to support restrictive approaches. These groups therefore represent important target audiences for public health campaigns designed to build support for the introduction of regulation relating to e-cigarettes. Few differences were found by gender and age, suggesting that campaigns do not need to be tailored to specific sociodemographic groups.

### Limitations

The present study should be interpreted in the context of its limitations. First, the use of a web panel resulted in a non-representative sample being obtained, particularly with respect to smoking status. As noted above, the present study comprised a greater proportion of smokers relative to the proportion observed in the NDSHS (25% vs. 17%). However, as smokers are significantly more likely to be e-cigarette users than are non-smokers [45, 46], canvassing their support for e-cigarette policies is important. Second, although this study examined support for a range of potential e-cigarette policies, many other policies are also worthy of exploration. For example, e-cigarettes are available in over 7700 unique flavours [47], and concerns have been raised that such flavours increase the appeal of e-cigarettes, particularly among youth populations [30, 48]. It has therefore been suggested that regulatory efforts should include restricting the availability of e-cigarette flavours [30, 38, 49]. Evidence also indicates that the ability to perform tricks with e-cigarette vapour is particularly appealing to youth [30, 50], and it has thus been proposed that e-cigarettes with visible vapours should be restricted [30]. Future research could seek to explore support for these kinds of more specific policies related to e-cigarette products.

## Conclusion

Regulatory action is needed to protect the health of users and non-users and ensure e-cigarettes do not contribute to the renormalisation of smoking and potentially undermine decades of public health efforts [6, 51, 52]. The present study provides initial insight into Australian young adults’ support for potential regulatory approaches to e-cigarettes. These results have implications for Australian policymakers at all governmental levels in terms of prioritising policy actions and developing strategies to increase support for specific policies among the population segment that is most likely to be using these products [53].

## Additional file

Additional file 1: Items used in the study “Support for e-cigarette regulations among Australian young adults”. (DOCX 28 kb)

## Abbreviations

NDSHS: National Drug Strategy Household Survey
