# Supplementary material for: Support for e-cigarette regulations among Australian young adults
Source: BMC Public Health. 2019 Jan 15;19:67. doi: 10.1186/s12889-019-6410-4 (PMC6334386; doi:10.1186/s12889-019-6410-4)
Supplement: Supplementary file 1 — Items used in the study “Support for e-cigarette regulations among Australian young adults”. (DOCX 28 kb) [file 12889_2019_6410_MOESM1_ESM.docx]

**Items used in the study “Support for e-cigarette regulations among Australian young adults”**

**Demographics**

What is your age?

What is your gender?

Male

Female

Please enter your postcode: ________________

**Tobacco use**

Which of the following products have you ever tried, even just one time? (select all that apply)

Tobacco cigarettes (including roll-your-own cigarettes)

Cigars, little cigars, or cigarillos

Chewing tobacco

Pipes for smoking tobacco

Hookah, shisha, or waterpipes

Snus

Dissolvable tobacco products (e.g., Ariva, lozenges, or other tobacco products that dissolve in the mouth)

None of these

Over your lifetime, would you have smoked at least 100 cigarettes or a similar amount of tobacco?

Yes

No

Don’t know or can’t say

Which of the following products have you used in the last 30 days? (select all that apply)

Tobacco cigarettes (including roll-your-own cigarettes)

Cigars, little cigars, or cigarillos

Chewing tobacco

Pipes for smoking tobacco

Hookah, shisha, or waterpipes

Snus

Dissolvable tobacco products (e.g., Ariva, lozenges, or other tobacco products that dissolve in the mouth)

**E-cigarettes use**

*The following questions are about electronic cigarettes (often referred to as e-cigarettes or e-cigs) only*

Have you ever heard of e-cigarettes?

Yes

No

Unsure

E-cigarettes are battery-powered cigarette-like tubes that illuminate or glow at the end and provide a vapour containing either nicotine or flavourings such as apple or coffee.

Have you ever used an e-cigarette, even just one or two puffs?

Yes

No

Have you used an e-cigarette in the last 30 days, even one or two puffs?

Yes

No

How frequently do you currently use e-cigarettes?

Daily

Weekly

Fortnightly

Monthly

Less than monthly

What are the reasons you first tried e-cigarettes?

Open response:

What are the reasons you currently use e-cigarettes?

Open response:

**E-cigarettes - health beliefs**

How harmful do you think e-cigarettes are to health?

1 Not at all harmful

2

3

4

5 Very harmful

Don’t know

Which of the following statements best describe how harmful you consider e-cigarettes compared to tobacco cigarettes?

E-cigarettes are less harmful than tobacco cigarettes

E-cigarettes and tobacco cigarettes are equally harmful

E-cigarettes are more harmful than tobacco cigarettes

Don’t know

**E-cigarettes - policy questions**

To what extent do you agree or disagree with the following statements?

|  | Strongly disagree | Disagree | Neither agree nor disagree | Agree | Strongly Agree | Don’t know |
| --- | --- | --- | --- | --- | --- | --- |
|  | 1 | 2 | 3 | 4 | 5 |  |
| You should be able to use e-cigarettes in places that do not allow smoking |  |  |  |  |  |  |
| E-cigarettes should be treated as if they are prescription medicines |  |  |  |  |  |  |
| E-cigarettes should be treated as if they are tobacco cigarettes |  |  |  |  |  |  |
| E-cigarettes should only be sold in pharmacies like other non-cigarette products that contain nicotine (e.g., patches, gums, lozenges) |  |  |  |  |  |  |
| E-cigarettes should be made readily available as an over the counter purchase at regular shops |  |  |  |  |  |  |
| The supply of e-cigarettes that contain nicotine should be prohibited |  |  |  |  |  |  |
| The supply of e-cigarettes that do not contain nicotine should be prohibited |  |  |  |  |  |  |
